# Genome-scale metabolic model of *Staphylococcus epidermidis* ATCC 12228 matches *in vitro* conditions

**DOI:** 10.1128/msystems.00418-25

**Published:** 2025-05-21

**Authors:** Nantia Leonidou, Alina Renz, Benjamin Winnerling, Anastasiia Grekova, Fabian Grein, Andreas Dräger

**Affiliations:** 1 Institute for Bioinformatics and Medical Informatics (IBMI), Eberhard Karl University of Tübingen, Tübingen, Germany; 2 Department of Computer Science, Eberhard Karl University of Tübingen, Tübingen, Germany; 3 German Center for Infection Research (DZIF), Tübingen, Germany; 4 Quantitative Biology Center (QBiC), Eberhard Karl University of Tübingen, Tübingen, Germany; 5 Division Systems Biology of Signal Transduction, German Cancer Research Center (DKFZ) 28333, Heidelberg, Baden-Württemberg, Germany; 6 Institute for Pharmaceutical Microbiology, University of Bonn 9374, Bonn, North Rhine-Westphalia, Germany; 7 German Center for Infection Research (DZIF), Bonn, Germany; 8 Structural and Computational Biology Unit, European Molecular Biology Laboratory (EMBL) 9471, Heidelberg, Baden-Württemberg, Germany; 9 Data Analytics and Bioinformatics, Institute of Computer Science, Martin Luther University Halle-Wittenberg, Halle (Saale), Germany; University of Rhode Island, Kingston, Rhode Island, USA

**Keywords:** genome-scale metabolic modeling, *S. epidermidis*, Gram positive, skin mcirobiota, nasal microbiota, systems biology, flux balance analysis, linear programming, SBML, FAIR principles

## Abstract

*Staphylococcus epidermidis*, a commensal bacterium inhabiting collagen-rich areas like human skin, has gained significance due to its probiotic potential in the nasal microbiome and as a leading cause of nosocomial infections. While infrequently leading to severe illnesses, *S. epidermidis* exerts a significant influence, particularly in its close association with implant-related infections and its role as a classic opportunistic biofilm former. Understanding its opportunistic nature is crucial for developing novel therapeutic strategies, addressing both its beneficial and pathogenic aspects, and alleviating the burdens it imposes on patients and healthcare systems. Here, we employ genome-scale metabolic modeling as a powerful tool to elucidate the metabolic capabilities of *S. epidermidis*. We created a comprehensive computational resource for understanding the organism’s growth conditions within diverse habitats by reconstructing and analyzing a manually curated and experimentally validated metabolic model. The final network, *i*Sep23, incorporates 1,415 reactions, 1,051 metabolites, and 705 genes, adhering to established community standards and modeling guidelines. Benchmarking with the Metabolic Model Testing suite yields a high score, indicating the model’s remarkable semantic quality. Following the findable, accessible, interoperable, and reusable (FAIR) data principles, *i*Sep23 becomes a valuable and publicly accessible asset for subsequent studies. Growth simulations and carbon source utilization predictions align with experimental results, showcasing the model’s predictive power. Ultimately, this work provides a robust foundation for future research aimed at both exploiting the probiotic potential and mitigating the pathogenic risks posed by *S. epidermidis*.

**IMPORTANCE:**

*Staphylococcus epidermidis*, a bacterium commonly found on human skin, has shown probiotic effects in the nasal microbiome and is a notable causative agent of hospital-acquired infections. While these infections are typically non-life-threatening, their economic impact is considerable, with annual costs reaching billions of dollars in the United States. To better understand its opportunistic nature, we employed genome-scale metabolic modeling to construct a detailed network of *S. epidermidis*’s metabolic capabilities. This model, comprising over a thousand reactions, metabolites, and genes, adheres to established standards and demonstrates solid benchmarking performance. Following the findable, accessible, interoperable, and reusable (FAIR) data principles, the model provides a valuable resource for future research. Growth simulations and predictions closely match experimental data, underscoring the model’s predictive accuracy. Overall, this work lays a solid foundation for future studies aimed at leveraging the beneficial properties of *S. epidermidis* while mitigating its pathogenic potential.

## INTRODUCTION

A prevalent constituent of the human skin flora is the coagulase-negative commensal (1, 2). This Gram-positive coccus predominantly inhabits the skin and mucosal membranes in areas such as the axillae, head, legs, arms, and nares. *Staphylococcus epidermidis* plays a crucial role in maintaining a balanced microbiome within the human nasal cavity, where harmful pathogens like *Staphylococcus aureus* commonly establish colonization. There is ongoing discourse regarding whether *S. epidermidis*, through competition in nutritionally scarce environments like the human nose, may exhibit probiotic effects against formidable pathogens such as *S. aureus* (2, 3). Nevertheless, *S. epidermidis* is recognized as a significant causative agent of nosocomial infections under specific conditions (4). It is particularly notable as the primary source of infections associated with indwelling medical devices, including intravascular catheters and implants such as prosthetic joints (1, 5, 6). The high occurrence of these nosocomial infections is attributed to *S. epidermidis*’s ubiquitous presence on the human skin, increasing the likelihood of contamination during the insertion of medical devices (7). Upon infection, *S. epidermidis* strains can form biofilms that protect them from antibiotics and host immune responses, making *S. epidermidis* infections highly resistant and challenging to eradicate (1, 7). Often, removing the foreign material becomes necessary to combat the infection effectively. While *S. epidermidis* infections seldom lead to life-threatening conditions, their impact on patients and the public health system is substantial. In the United States alone, the annual economic burden of *S. epidermidis* vascular catheter-related bloodstream infections is estimated to be around $2 billion (1, 6). Besides biofilm formation, *S. epidermidis* becomes pathogenic through several additional mechanisms that enable it to evade the immune system. These include the production of phenol-soluble modulins (PSMs) and extracellular proteases, which impair immune cell function and degrade immune components (8). *S. epidermidis* adheres to surfaces via surface-associated proteins, such as the Aap, facilitating colonization on medical devices (9). The bacterium also produces exopolysaccharides that protect it from immune detection, while its ability to acquire antibiotic resistance genes, especially against methicillin, allows it to survive treatment and establish chronic infections. Furthermore, it releases cytotoxins that damage host tissues and can acquire new virulence factors through horizontal gene transfer (10). Therefore, there is an urgent need for a more comprehensive understanding of *S. epidermidis* and its opportunistic characteristics to identify novel therapeutic strategies (1, 7).

One effective way to better understand and analyze an organism’s lifestyle and capabilities is through the reconstruction and analysis of genome-scale metabolic models (GEMs). These models rely on the organism’s annotated genome sequence, with genes encoding proteins of metabolic significance being linked to their respective reactions through gene-protein-reaction associations (GPRs). The resulting network integrates biochemical reactions and their associated metabolites with enzyme-coding genes specifying the catalytic activities of these reactions. Such models enable a comprehensive understanding of an organism’s metabolism at a systems level. Díaz Calvo et al. reconstructed the metabolic network of RP62A, a slime-producing and methicillin-resistant biofilm-forming isolate (11), while Guil et al. further validated it (12). However, the model lacks annotations and standardization in the identifiers for reactions and metabolites and does not include any associated genes. Additionally, we observed inconsistencies between the content of the model uploaded in the BioModels database (13) and the model provided in the supplementary material accompanying the manuscript.

**Fig 1:**
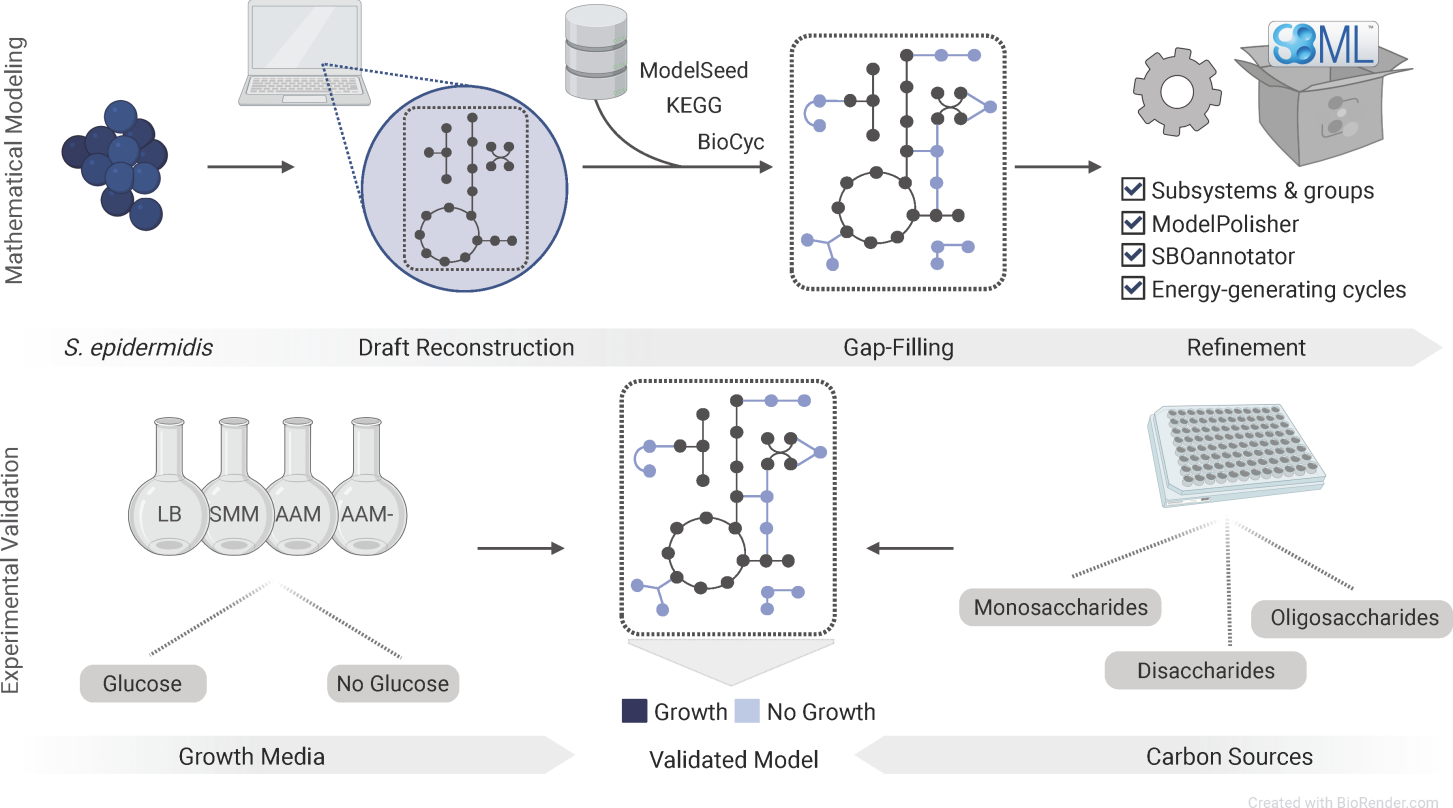
Reconstruction of a new metabolic network for *S. epidermidis* ATCC 12228, called *i*Sep23. The computational metabolic network was created and validated using a two-phase approach. The initial phase encompassed the mathematical representation of the metabolism using genome-scale models. In the second phase, the model underwent functional validation based on experimental data.

Here, we introduce *i*Sep23, a new manually curated, experimentally validated, and publicly available GEM, designed for the non-biofilm-forming *S. epidermidis* ATCC 12228. Figure 1 summarizes the computational and experimental approaches used in this study. The model comprises 1,415 reactions, 1,051 metabolites, and 705 genes and is freely available in the BioModels database (13) with the accession identifier MODEL2012220002. Moreover, it aligns with current community standards (14–16) and modeling guidelines (17, 18). Semantic benchmarking was conducted using the Metabolic Model Testing (MEMOTE) suite (19). Finally, *i*Sep23 follows the findable, accessible, interoperable, and reusable(FAIR) data principles (20), rendering it a valuable resource for subsequent research (21, 22). Growth simulations in various media were compared against laboratory experiments to assess the predictive capacity of the model. The model’s predictions regarding the utilization of diverse carbon sources were cross-referenced with experimental findings. Altogether, our model establishes a foundation for improved comprehension of the organism’s phenotypes and behavior under different nutritional conditions.

## RESULTS

### Properties of the constructed GEM

**Fig 2:**
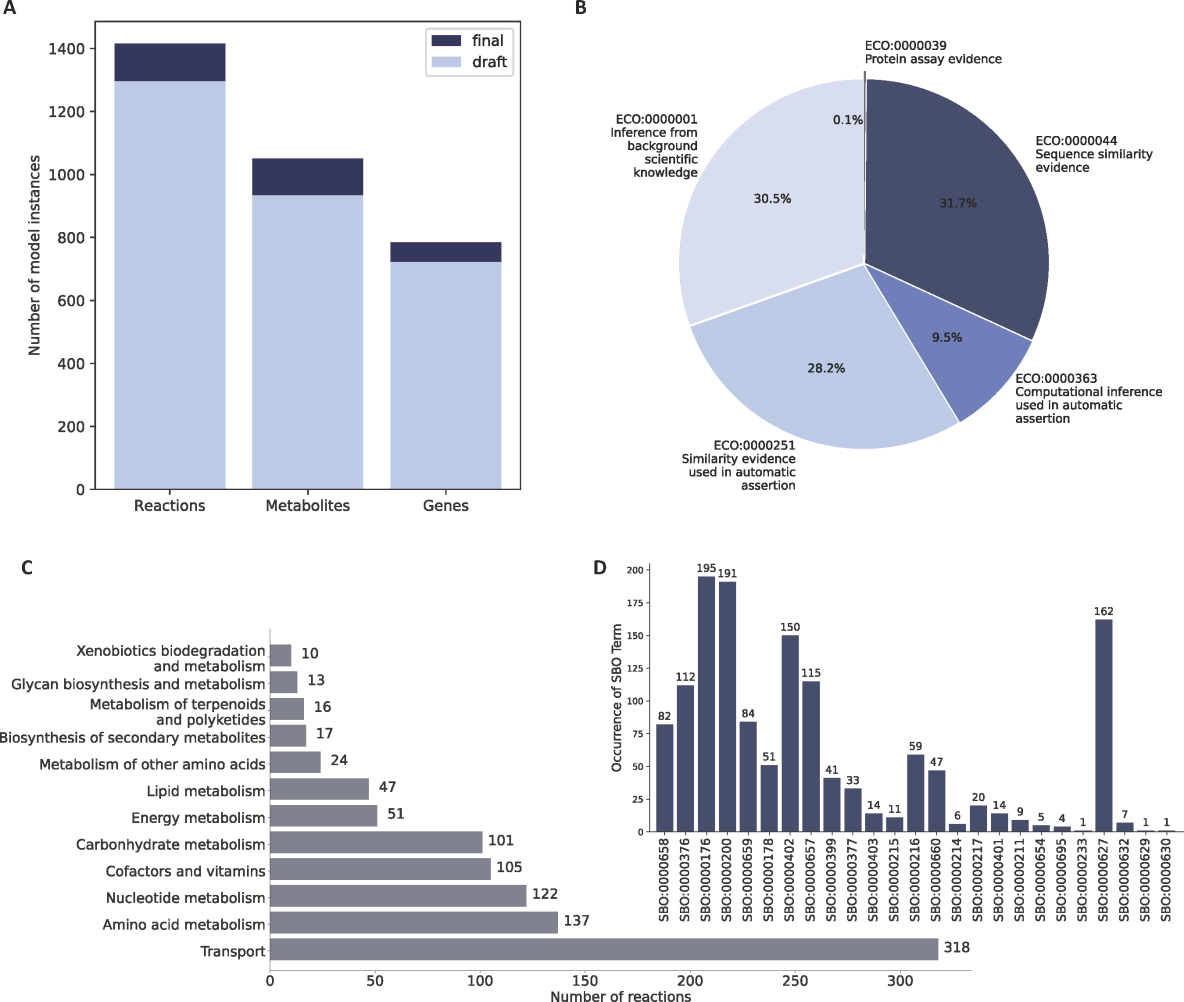
Reconstruction of a new metabolic network for properties of the network reconstructed for *S. epidermidis* ATCC 12228. (**A**) Quantitative comparison content between initial draft and final models. The draft network consisted of 1,295 reactions, 933 metabolites, and 722 genes. Further refinement and augmentation yielded the final metabolic model, comprising 1,415 reactions, 1,051 metabolites, and 785 genes. (**B**) Distribution of evidence and conclusion ontology (ECO) terms within the network. To characterize the inclusion evidence of biochemical reactions, ECO terms were assigned based on the associated GPR and UniProt evidence. The terms were allocated according to varying levels of evidentiary support. (**C**) Metabolic subsystem distribution of reactions. Each bar represents a distinct metabolic subsystem, with the length of the bar corresponding to the number of reactions in that pathway. (**D**) Coverage of systems biology ontology (SBO) terms within the metabolic network after applying the SBOannotator (24).

The initial draft model was built with CarveMe (23) and comprised 1,295 reactions, 933 metabolites, and 722 genes, yielding a MEMOTE score of 36%. Subsequent manual refinement involved the addition of 120 reactions, 118 metabolites, and 63 genes, as illustrated in Fig. 2A. This model represents the most comprehensive available reconstruction of *S. epidermidis*, exhibiting greater metabolic coverage with an increased number of reactions, metabolites, and associated genes compared to the RP2A model (Table 1). More specifically, the model was enhanced by adding key reactions, including tetrapeptide L,D-carboxypeptidase and various acyl-CoA dehydrogenases. Transport mechanisms were improved with the inclusion of uptake systems for L-cysteine, D-arabinose, glucose, and several amino acids (e.g., L-phenylalanine and L-tyrosine). Additionally, glycosyl transferases, glycerol-3-phosphate acyltransferase, and phosphatidylglycerol transport enzymes were incorporated to enhance lipid metabolism pathways. The model also includes reactions for iron transport (e.g., Fe-enterobactin and ferrioxamine G) and nucleotide metabolism (e.g., purine-nucleoside phosphorylases), significantly improving its capacity to simulate metabolic functions and nutrient utilization (Table S2). This represents the most comprehensive metabolic model available for *S. epidermidis*, as it includes remarkably more reactions, metabolites, and genes compared to the previously existing network for RP62A (11). Additionally, *i*Sep23 comprises over 980 GPRs and defines three distinct cellular compartments (cytosol, periplasm, and extracellular space). The 63 mass- and charge-imbalanced reactions initially found in the draft model were reduced to one mass-imbalanced and nine charge-imbalanced reactions, resulting in MEMOTE balance scores of 99.7% and 99.3%, respectively. Based on the literature evidence, we corrected the directionality of 34 enzymatic reactions in the model to ensure proper constraints during model simulations. Moreover, the final metabolic network does not include infeasible energy-generating cycles (EGCs) that could inflate the simulation results (see Materials and Methods). We annotated the model entities with cross-references to various databases and additional information to increase the model’s interoperability and re-usability. The reaction annotations are divided into three different biological qualifier types:

**TABLE 1:** Comparison of GEMs of *S. epidermidis^a^*

Parameter	*i*Sep23 (this study)	RP62A (11)	RP62A (BioModels [11])	RP62A (Guil et al. [12])
Reactions	1,415	1,064	1,065	990
Metabolites	1,051	938	939	864
Genes	785	0	0	0
Gene-protein-reaction rules	984	0	0	0
Defined subsystems	☑	□	□	□

^*a*^ The number of reactions, metabolites, genes, GPRs, and the presence of defined subsystems are considered. The model developed in this study exhibits the highest metabolic coverage, with a greater number of reactions, metabolites, and genes compared to the existing RP62A models. Due to discrepancies observed between the model uploaded to BioModels and the version provided in the supplemental material of the corresponding paper, both versions were considered in this analysis. A checked box means the presence of a feature, whereas an empty box denotes its absence.

- BQB_IS_DESCRIED_BY: stores ECO terms, which provide evidence supporting the reaction.
- BQB_IS: stores cross-references to eight external databases, linking the reaction to corresponding entries.
- BQB_OCCURS_IN: stores information about pathways associated with biochemical reactions.

**Fig 3:**
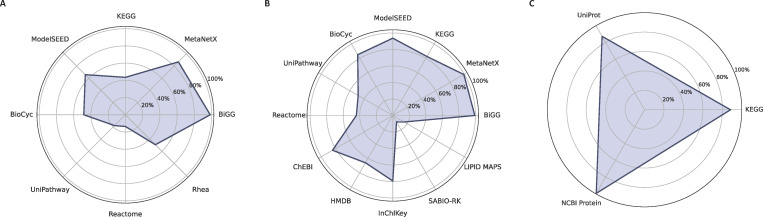
Reconstruction of a new metabolic network for cross-references incorporated into the metabolic network *i*Sep23. Each axis represents a different cross-reference source, with the plotted values indicating the percentage of entries for (**A**) reactions, (**B**) metabolites, and (**C**) genes. The chart highlights the relative abundance of various cross-reference sources in the metabolic network.

The inclusion of ECO terms ensures a comprehensive understanding of evidence and assertion methodologies (25), thereby facilitating robust quality control measures and evidence queries. The ECO term with the lowest evidence level is ECO:0000001, coding for inference from background scientific knowledge. This term was ascribed to 30.2% of the biochemical reactions within the network (Fig. 2B). Notably, this percentage encompasses pseudoreactions, such as exchanges, sinks, demands, and biomass function. Within the group of 431 reactions associated with this ECO term, 170 pertained to pseudoreactions. The ECO term ECO:0000251 denotes similarity evidence used in automatic assertion and was assigned to 28.5% of all reactions. Moreover, the terms ECO:0000251 (computational inference used in the automatic assertion) and ECO:0000044 (sequence similarity evidence) annotated 9.3% and 31.9% of all reactions, respectively. A minimal fraction (0.1%) of reactions exhibits protein assay evidence, identified by the ECO:0000039 term. In *i*Sep23, transporters are the most frequent subsystem with 318 reactions, highlighting their importance in nutrient exchange. Amino acid metabolism (137 reactions) and nucleotide metabolism (122 reactions), essential for protein and nucleic acid synthesis, are also prominent (Fig. 2C). Less frequent subsystems include the metabolism of terpenoids and polyketides (16 reactions) and xenobiotics biodegradation and metabolism (10 reactions), indicating a more limited role in detoxification and specialized biosynthetic pathways. Subsequently, the SBOannotator was utilized to annotate the model with precise and descriptive SBO terms (24) (Fig. 2D). In total, 25 terms describing classes of various model elements were incorporated. Finally, similar to reactions, all metabolites and genes were annotated with 12 and 3 additional external databases, respectively, using the biological qualifier type BQB_IS (Fig. 3). These modifications resulted in the final metabolic network, with an overall MEMOTE score of 88. In contrast, the metabolic network for RP62A has a MEMOTE score of only 14% and lacks both GPRs and cellular compartments.

The final curated metabolic model was stored as a Systems Biology Markup Language (SBML) (26) file. This format supports the integration of various plugins, such as the fbc package (27) and the groups (28), both of which are enabled in *i*Sep23. The group package allows for incorporating additional information without altering the mathematical interpretation of the model. We defined all pathways and subsystems identified from the Kyoto Encyclopedia of Genes and Genomes (KEGG) database (29) as individual groups and assigned the corresponding reactions as members. Overall, we added 99 distinct groups to the model that facilitate pathway-related analysis.

### Validation of the metabolic network

In addition to syntactic evaluation, data structure, and file format validation, the model was assessed for its predictive value by comparing simulation outcomes with empirical laboratory data. Given the adaptability of microbes to diverse environments, we focused on investigating their growth behavior across various nutrient media, ensuring that simulated conditions closely matched the experimental setups.

#### Evaluation of different growth media

First, we conducted growth simulations using various chemically defined media. Specifically, we utilized three synthetic minimal media: synthetic minimal medium (SMM) (30), AAM (31), and AAM− (32). Developed initially to explore the metabolic requirements of *S. aureus*, these media definitions served as the basis for our simulations. We used the compound concentrations specified in the media definitions for the *in silico* simulations and tested whether our model exhibited growth under these conditions. Furthermore, we extended our evaluations to include the widely used lysogeny broth (LB) medium. Through a combination of *in silico* and *in vitro* experiments, we investigated the growth behavior in four different media, both with and without D-glucose as the carbon source. This dual approach allowed us to measure growth in a simulated environment and in a real laboratory setting, providing a comprehensive validation of the model’s predictive performance under various nutritional conditions. Additionally, we refined our metabolic network based on experimental outcomes by adding necessary reactions and eliminating those lacking genetic evidence. During this manual model curation, we incorporated reactions to improve alignment with experimental data, including biotin and cysteine transporter and exchange reactions, as well as transporters and exchange reactions for L-phenylalanine, L-arginine, and L-tryptophan. Additionally, a nicotinamide uptake reaction was incorporated to refine the model’s metabolic capabilities further. Conversely, transport reactions via the phosphoenolpyruvate and pyruvate phosphotransferase system for D-glucosamine, D-mannose, mannitol, and cellobiose were removed due to insufficient genetic evidence (Table S2).

**Fig 4:**
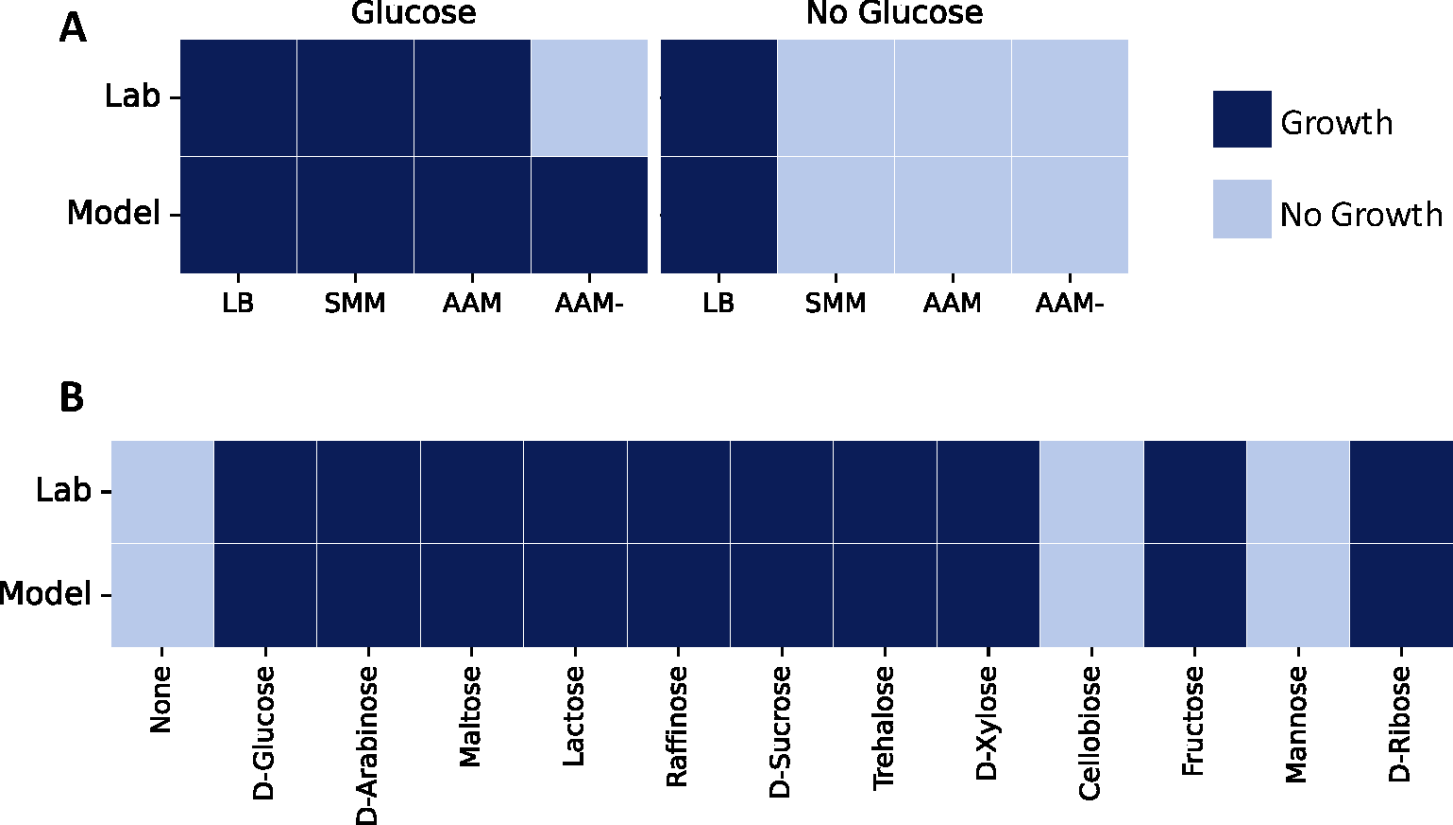
Reconstruction of a new metabolic network for growth phenotypes of *S. epidermidis* in different nutritional environments. (**A**) Evaluation of *S. epidermidis* growth encompassed various environmental conditions, including testing on complete LB and three minimal media formulations: SMM, AAM, and AAM−, both with and without D-glucose as a carbon source. (**B**) Analysis of growth in different carbon sources utilized SMM as the primary medium, where different amounts of other sugars systematically replaced glucose. Simulation results closely paralleled laboratory findings, ensuring consistency across computational predictions and experimental outcomes.

**Fig 5:**
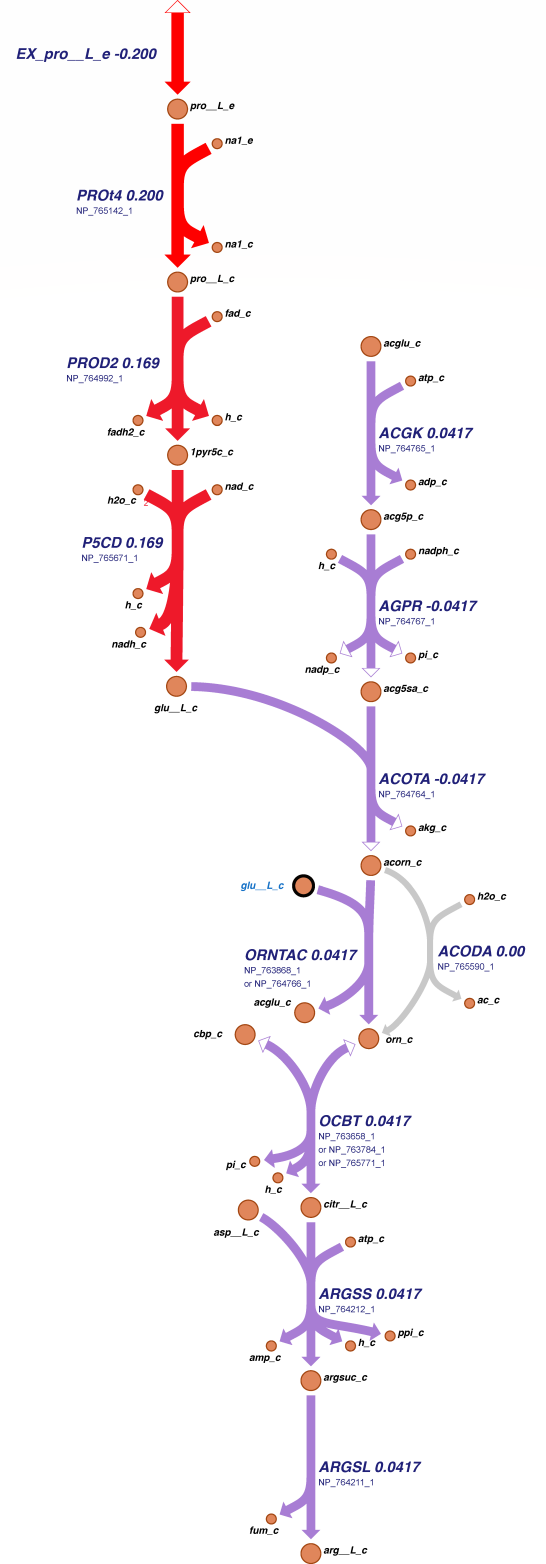
Reconstruction of a new metabolic network for the biosynthetic pathway of L-arginine via L-glutamate and L-proline in *i*Sep23, extracted from biochemical databases. The colors represent the magnitude of the simulated reaction fluxes in the model. Gray arrows indicate zero flux, while colored arrows represent non-zero fluxes in AAM−. All available L-proline is actively taken up and subsequently metabolized into various products, including L-glutamate, L-ornithine, and ultimately L-arginine. Genetic evidence supporting each reaction is provided as gene-reaction rules within *i*Sep23. Figure created with Escher (35).

Figure 4 illustrates the growth behavior of *S. epidermidis* in various environments both *in silico* and *in vitro*. The computational model successfully simulated growth across all media when glucose was utilized as the sole carbon source. However, growth was observed exclusively in LB without glucose (Fig. 4A). On the contrary, *in vitro* experiments revealed no growth in AAM−, a medium lacking L-arginine. Comparative analysis of AAM−, AAM, and SMM highlights the absence of L-arginine in AAM−, a compound crucial for *S. epidermidis* growth. Prior studies have identified L-arginine auxotrophies in *Staphylococcus* species, including *S. epidermidis* (33). Despite reported L-arginine auxotrophy, the *S. epidermidis* strain ATCC 12228 harbors biosynthetic pathways for L-arginine via L-ornithine and L-glutamate, as reported in BioCyc (34) and KEGG (29). In AAM−, L-glutamate is not provided as an amino acid, but it can be synthesized from L-proline, an amino acid present in the medium. The biosynthetic pathway as derived from the databases is illustrated in Fig. 5. All available L-proline is taken up and metabolized into several products, including L-glutamate, L-ornithine, and L-arginine. Each reaction in this pathway is supported by genetic evidence through a GPR within *i*Sep23.

#### Growth in different carbon sources

In addition to evaluating *S. epidermidis*’s growth behavior in different media, we assessed the utilization of various carbon sources. This involved employing SMM and substituting D-glucose with alternative sugars in amounts adjusted for carbon content. A total of 12 different sugars were subjected to evaluation, as illustrated in Fig. 4B. Except for cellobiose and D-mannose, *S. epidermidis* demonstrated the capability to utilize all tested sugars as a carbon source, both through computational simulations (*in silico*) and laboratory experiments (*in vitro*). This consistency between model predictions and experimental observations lends robust support to the accuracy of the computational model.

## DISCUSSION

Here, we present a manually curated GEM of *S. epidermidis* ATCC 12228, *i*Sep23. Literature-based corrections and meticulous manual curation ensured the accurate representation of enzymatic reaction directions, which is essential for precise constraints during simulations. Overall, our model aligns with experimental data and offers a comprehensive platform for exploring *S. epidermidis*’s metabolic capabilities and behavior under diverse conditions. Exploring the growth patterns under varied nutritional environments provides valuable insights into activated metabolic pathways, enriching our comprehension of the bacterium’s adaptability across diverse conditions. This understanding is pivotal for predicting bacterial behavior and survival strategies in specific niches. Additionally, discerning the impact of specific nutrients in host environments on bacterial virulence and infection establishment is essential, guiding the development of strategies to control or inhibit bacterial pathogens. The growth media tested in this work simulate nutrient conditions relevant to environments where *S. epidermidis* infections occur. SMM and AAM provide essential nutrients for minimal growth, reflecting nutrient-limited conditions. In contrast, LB offers a nutrient-rich profile, mimicking more favorable niches. In the human host, sugars and other carbon sources are available primarily from dietary carbohydrates, which are broken down into simpler sugars during digestion. The primary sugar in human blood is glucose, produced in the liver and released into circulation, making it the most abundant free carbohydrate in human serum (36). The inconsistency between the *in silico* and *in vitro* results regarding the AAM− in the presence of glucose could be attributed to factors beyond the metabolic scope. For instance, non-metabolic factors could be regulatory mechanisms and post-translational modifications. *Staphylococcus* sp. have reported auxotrophies for various amino acids, including arginine (33). However, recent studies suggest that this is due to condition-specific regulatory mechanisms (carbon catabolite repression) rather than a permanent loss of biosynthetic capability. These mechanisms repress arginine synthesis in response to glucose, particularly when proline is the substrate (37) but not when glutamate is used (38). Sadykov demonstrated that the carbon catabolite-responsive regulator *CcpA* plays a critical role in biofilm formation and virulence by inactivating the *CcpA* gene in *S. epidermidis* 1457 (39). The observed discrepancy highlights the need for a deeper understanding of the regulatory and metabolic factors influencing *S. epidermidis* growth in AAM−, with experimental validation crucial to resolving the difference between *in silico* and *in vitro* outcomes.

All in all, the refined network serves as a powerful tool for exploring *S. epidermidis*’s metabolic capabilities and behavior under diverse conditions. Future perspectives involve leveraging the model for targeted studies, such as investigating metabolic pathways, assessing the impact of genetic modifications, and exploring potential drug targets. The model’s compatibility with the fbc and groups packages in the SBML level 3 version 1 (16) format enhances its flexibility, enabling the integration of additional plugins for more intricate analyses. Including 99 distinct groups representing pathways and subsystems from the KEGG database provides a foundation for comprehensive pathway-related analyses. Altogether, *i*Sep23 aligns with experimental data and lays the groundwork for future investigations into the bacterium’s metabolism. Its accuracy, comprehensibility, and flexibility make it a valuable resource for advancing our understanding of microbial physiology and metabolic engineering applications.

## MATERIALS AND METHODS

### Reconstruction and manual refinement of the metabolic network

The reconstruction of the presented GEM is based on protocols described in previous studies (40, 41). The fast and automated reconstruction tool CarveMe (23) curates genome-scale metabolic models of microbial species and communities (23). During the initial curation phase, a universal model was systematically compared to the annotated genome sequence of the species of interest, facilitating the construction of individual single-species metabolic models. In this study, we utilized CarveMe version 1.2.2 and the annotated genome sequence of *S. epidermidis* ATCC 12228 with the RefSeq (42) accession ID NC_004461.1 that covers the bacterial chromosome. Throughout the drafting process and subsequent model iterations, rigorous monitoring and benchmarking were conducted using MEMOTE and the SBML Validator from libSBML (19, 43). MEMOTE performs standardized semantic tests across four key domains: annotation, basic tests, biomass reaction, and stoichiometry. The results are stored in a comprehensive report that includes the model’s overall performance assessed by a metric called MEMOTE score (denoted as a percentage with 100%). A higher MEMOTE score correlates with enhanced annotation quality, greater consistency, and formal correctness of the model in SBML (26) format. We utilized the SBML Validator to check the model file for syntax errors such as improper structure, incorrect tags, missing attributes, and invalid values, ensuring a valid model format. To refine the initial model automatically, the ModelPolisher (44) was employed in a preliminary step. Leveraging the Biochemical, Genetical, and Genomical (BiGG) database (45), identifiers of the model entities, ModelPolisher systematically accessed the BiGG database, assimilating all available information for these entities into the network as annotations.

In the initial draft model, a total of 63 reactions were identified as exhibiting mass and charge imbalances. To address the identified imbalances, the MassChargeCuration tool was utilized to correct mass- and charge-imbalanced reactions (46). Databases such as MetaNetX (47) and BioCyc (34) were consulted to obtain accurate information regarding the charges and chemical formulas of the metabolites involved in these reactions. This approach enabled precise adjustments to the mass and charge imbalances within the model, achieving 100% stoichiometric consistency and over 95% mass and charge balance.

Additionally, the network constraints were carefully reviewed. Enzymes frequently act as catalysts in metabolic reactions. However, some enzymes effectively catalyze the reaction only in one direction. Consequently, it becomes imperative to impose constraints on the directionality of a given reaction. Cases where irreversible reactions are erroneously modeled as reversible can result in an artificial expansion of the solution space within simulations. Conversely, misrepresenting reversible reactions as irreversible can unduly constrict the solution space, thereby precluding potential solutions. During our analysis, we systematically assessed various reaction directionalities and rectified any inaccuracies as needed, using information derived from the organism-specific BioCyc database.

#### Model extension

The model extension involved the integration of supplementary reactions sourced from established literature. The knowledge bases utilized for this purpose included BioCyc (34), KEGG (29), and ModelSEED (48). To identify relevant genetic information, locus tags from gene annotations were extracted and compared against the KEGG database. Reactions catalyzed by hypothetical enzymes were excluded from the analysis. Candidate reactions were systematically cross-referenced with the BiGG (45) and ModelSEED databases and were subsequently integrated into the network with BiGG identifiers and corresponding GPRs. If no entry in the BiGG database was specified, reaction identifiers from the source database were used.

#### Detecting energy-generating cycles

GEMs with EGCs may harbor thermodynamically inaccurate cycles capable of generating energy without concurrent nutrient consumption (49). These undesirable loops necessitate detection and subsequent elimination from the model. Fritzemeier et al. developed a systematic workflow for different energy metabolites. A dissipation reaction was introduced into the model for each energy metabolite. After the imposition of constraints whereby all uptake rates were set to zero, an optimization process was conducted on the dissipation reaction. The presence of a non-zero flux following optimization serves as an indicator of the existence of EGCs within the model.

#### Including gene annotations

The software ModelPolisher (44) was used to annotate the model entities. It is noteworthy, however, that this tool does not facilitate the annotation of model genes due to their strain-specific nature. We annotated the network genes using the associated National Center for Biotechnology Information (NCBI) protein identifiers (50). Notably, these gene identifiers underwent modifications during reconstruction due to the prokaryotic RefSeq genome re-annotation project (42). To address this, we retrieved the updated NCBI protein identifiers from the NCBI database (50). Subsequently, leveraging these novel protein identifiers in conjunction with the organism’s GenBank file (51), we extracted the corresponding KEGG gene identifiers, which align with the organism’s locus tag and UniProt identifiers (52). The integration of cross-references was executed as annotations using libSBML (43). This comprehensive process ensures the accuracy and coherence of gene annotations within the model, thereby contributing to the reliability and accuracy of subsequent analyses.

#### Adding subsystems and groups

The reaction-associated pathways were retrieved using the annotated KEGG identifiers and the KEGG REST API. Subsequently, these pathways were incorporated as annotations utilizing the biological qualifier BQB_OCCURS_IN. Furthermore, the group’s package was activated for enhanced functionality. Each identified pathway was integrated as a group and the corresponding reactions as members.

#### Adding ECO and SBO terms

To enhance the model’s reusability, we incorporated ECO terms that annotate all metabolic reactions (25). This ontology comprises terms and classes of the various evidence and assertion methods. These terms elucidate, for instance, the nature of evidence associated with a gene product or reaction, facilitating robust model quality control. The assignment of a suitable ECO term to each reaction involved the extraction of GPRs. If a reaction lacked a GPR, the term ECO:0000001 was ascribed, denoting its inference from background scientific knowledge. Conversely, for all reactions with a GPR, the protein’s existence was reviewed in the UniProt database (52). We distinguished the presence of proteins based on distinct categories, namely: (i) inferred from homology (ECO: 0000044), (ii) predicted (ECO: 0000363), (iii) evidence at the transcript level (ECO: 0000009), or (iv) protein as say evidence. Genes not found in UniProt were assigned the term ECO:0000251, indicating the similar evidence used in an automatic assertion. The relevant ECO term was incorporated as an annotation in cases where a biochemical reaction was associated with a GPR described by a single gene. If the GPR involved multiple genes, the gene associated with the lowest evidence score was appended. All ECO terms were supplemented with the biological qualifier BQB_IS_DESCRIBED_BY.

The SBOannotator (24) was employed to assign SBO terms to all reactions, metabolites, and genes within the metabolic network. These terms offer unambiguous semantic information, delineating the type or role of each model component.

#### Elimination of redundant information

CarveMe stores the annotation information on model entities and cross-references to external databases within the notes field. However, the annotation field in the form of the controlled vocabulary terms is more appropriate for this information. Hence, we transferred all cross-references to the annotation field using the ModelPolisher (44). Subsequently, the annotation information was systematically removed from the notes field to optimize file size and eliminate redundancy in information storage.

### Formulation of the linear programming framework

We employed constraint-based modeling, specifically flux balance analysis (FBA), to determine flux distribution through the optimization of an objective function using linear programming (53). The metabolic network is mathematically encoded in a stoichiometric matrix S, which maps the connectivity of mass- and charge-balanced reactions to metabolites. At steady state, the system of linear equations derived from the network is defined as follows:


(1) $$\begin{array}{lcr} & S\cdot \vec{v}=0 & \end{array}$$

with S being the stoichiometric matrix and $\vec{v}$ being the flux vector. Constraints are imposed to restrict the solution space and ensure biological relevance. Altogether, the FBA maximization problem, incorporating mass balance, thermodynamic, and capacity constraints, is formulated as follows:


(2) $$\begin{array}{ll} \mathrm{maximize} & Z=c^{T}\vec{v}\\ \text{ subject to: } & S\cdot \vec{v}=\vec{0}\\ & v_{\min}\leq v_{r}\leq v_{\max}\;\text{ for }r\in \{1,\ldots ,n\}\\ & \forall r\in I:0\leq v_{r} \end{array}$$

Here, n is the amount of reactions, Z represents the linear objective function, and $\vec{c}$ is a vector of coefficients on the fluxes $\vec{v}$ used to define the objective function.

### Evaluation and validation of growth capabilities

#### Different growth media

The growth behavior of *S. epidermidis* was assessed in three distinct synthetic minimal media initially formulated to investigate the metabolic requirements of *S. aureus*. These are the (i) SMM (30), (ii) AAM (30), and (iii) AAM− (32); a modified version of the AAM medium. The concentrations of the various components served as lower bounds for the corresponding exchange reactions of metabolites, as detailed in Table S1. In addition to the already provided salts and ions, we added minimal traces of zinc (EX_zn2_e), cobalt (EX_cobalt2_e), and copper (EX_cu2_e) to the simulated medium to enable growth. The lower bound of these reactions was set to −0.0001 mmol/(gDW⋅h). Oxygen availability was defined by setting the lower bound of the exchange reaction to −20 mmol/(gDW⋅h). The initial formulation of the three media involved the use of nicotinic acid. However, as nicotinic acid was substituted with nicotinamide in laboratory experiments, our simulated media also incorporated nicotinamide. In addition to the three minimal media, we tested *S. epidermidis*’s growth on the LB (23). The lower bounds of the compounds’ exchange reactions listed in Table S1 were set to −10 mmol/(gDW⋅h). All *in silico* simulations were evaluated with and without D-glucose as a carbon source.

#### Different carbon sources

Twelve different sugars were tested for their potential role as a carbon source: D-glucose, D-arabinose, maltose, lactose, raffinose, D-sucrose, trehalose, D-xylose, D-cellobiose, fructose, mannose, and D-ribose. For the growth simulations in different carbon sources, we used the SMM with nicotinamide instead of nicotinic acid as a basis (Table 2). The concentrations reported in the medium were established as lower bounds for the simulation. The concentrations of the listed carbon sources were calculated to be equivalent in carbon content to the initial 5 g/L of glucose used in the defined SMM.

**TABLE 2:** Testing of different carbon sources^*a*^

Sugar	Reaction ID	Lower bound
D-glucose	EX_glc__D_e	−5.00
D-arabinose	EX_arab__L_e	−4.15
Maltose	EX_malt_e	−9.48
Lactose	EX_lcts_e	−9.48
Raffinose	EX_raffin_e	−13.97
D-sucrose	EX_sucr_e	−9.48
Trehalose	EX_tre_e	−9.48
D-xylose	EX_xyl__D_e	−4.15
D-cellobiose	EX_cellb_e	−9.48
Fructose	EX_fru_e	−5.00
Mannose	EX_man_e	−5.00
D-ribose	EX_rib__D_e	−4.15

^*a*^ Twelve different sugars were tested for their potential to serve as a carbon source in *S. epidermidis*. All values are given in mmol/(gDW·h).

### Laboratory validation

#### Media preparation

The minimal media AAM, AAM−, and SMM were prepared as carbon-source-free base media following the methods provided by Machado et al. after omitting glucose as the default carbon source (30). The carbohydrates that replaced glucose as alternative carbon sources were dissolved in their respective base medium, and the resulting media were sterile filtered. Carbohydrates were obtained from Carl Roth (D-arabinose, D-glucose, trehalose, lactose, sucrose, and raffinose), EMD-Millipore (fructose), Fluka (maltose, D-cellobiose), and Sigma Aldrich (mannose, D-ribose, and D-xylose) in purity grades of ≥98. LB was prepared following the standard formulation of 10 g/L tryptone (MP Biomedicals), 10 g/L sodium chloride (Carl Roth), 5 g/L yeast extract (Carl Roth), and 5 g/L glucose when required.

#### Growth experiments

Cultures of *S. epidermidis* ATCC 12228 were initiated by inoculating overnight precultures in LB at 37°C. Subsequently, primary cultures in LB were established from them and allowed to grow to an optical density (OD) at 600 (OD_600_) of 0.5. Cell harvesting was achieved through centrifugation and two washes with the carbon-source-free medium. The cells were then resuspended to an OD_600_ of 0.05 in media containing the respective carbon source. Growth was assessed by determination of the OD after a 24-hour incubation at 37°C. Growth experiments were performed in at least three biological replicates in a 96-well plate format. OD measurements were performed with a Tecan Spark microplate reader.

## Data Availability

Supplemental data are available along with this article. Additionally, *i*Sep23 is available at the BioModels Database (13) as an SBML Level 3 Version 1 (16) file. Access the model at https://www.ebi.ac.uk/biomodels/MODEL2012220002.

## SUPPLEMENTAL MATERIAL

Table S1 msystems.00418-25-s0001.xlsx Media composition.

Table S2 msystems.00418-25-s0002.xlsx Model changes.

ASM does not own the copyrights to Supplemental Material that may be linked to, or accessed through, an article. The authors have granted ASM a non-exclusive, world-wide license to publish the Supplemental Material files. Please contact the corresponding author directly for reuse.
